# High brucellosis prevalence and risk among children aged 5–14 years in Kenyan pastoral health facilities: A facility-based surveillance study

**DOI:** 10.1371/journal.pntd.0014129

**Published:** 2026-08-10

**Authors:** Dismas C. O. Oketch, Ruth Njoroge, Isaac Ngere, John Gachohi, Walter Jaoko, Samuel Waiguru Muriuki, Athman Juma Mwatondo, Konongoi S. Limbaso, Mathew Muturi, Jodie Withall, John Mwaniki Njeru, Boru Ali, Boku Bodha, Lydia Kilowua, Nazaria Wanja Nyaga, Moshe Alando, David Maina, Samoel Ashimosi Khamadi, M. Kariuki Njenga, Roland T. Ashford, Eric Mogaka Osoro

**Affiliations:** 1 Washington State University Global Health Program, Nairobi, Kenya; 2 Department of Medical Microbiology and Immunology, University of Nairobi, Nairobi, Kenya; 3 Paul G. Allen School for Global Health, Washington State University, Pullman, Washington, United States of America; 4 School of Public Health, Jomo Kenyatta University of Agriculture and Technology, Nairobi, Kenya; 5 Department of Biological Sciences, University of Idaho, Moscow, Idaho, United States of America; 6 Zoonotic Disease Unit, Government of Kenya, Nairobi, Kenya; 7 Kenya Medical Research Institute, Centre for Virus Research, Nairobi, Kenya; 8 Department of Veterinary Medicine, Dahlem Research School of Biomedical Sciences (DRS), Freie Universität Berlin, Berlin, Germany; 9 Department of Bacteriology, Animal and Plant Health Agency (APHA), Weybridge, United Kingdom; 10 Centre for Microbiology Research, Kenya Medical Research Institute, Centre for Microbiology Research, Nairobi, Kenya; 11 County Government of Marsabit, Marsabit, Kenya; 12 County Government of Kajiado, Kajiado, Kenya; Colorado State University, UNITED STATES OF AMERICA

## Abstract

**Background:**

Brucellosis remains endemic in pastoral sub-Saharan Africa, but facility-based data on human prevalence and associated factors in these communities are limited. We estimated brucellosis prevalence and associated factors among febrile patients at two pastoral health facilities in Kenya.

**Methodology/Principal findings:**

We conducted prospective facility-based surveillance from February 2023 to July 2024 at Laisamis Sub-County Referral Hospital, Marsabit County, and Mailwa Health Center, Kajiado County. Patients aged ≥1 year with acute febrile illness and brucellosis-compatible symptoms were enrolled consecutively. Blood samples were tested using Rose Bengal Test serology, real-time PCR for *Brucella* DNA, and indirect ELISA. Cases were classified hierarchically as RBT titer ≥1:8, PCR positivity among low-titer RBT-reactive samples, or low-titer RBT reactivity with ELISA IgG positivity. Modified Poisson regression with robust variance estimated adjusted prevalence ratios (aPR). Of 443 enrolled participants, four lacked sufficient laboratory information, leaving 439 for analysis. Overall, 67 participants met the brucellosis case definition (15.3%; 95% CI: 12.2–18.9%). Prevalence was higher in Laisamis than Mailwa (19.4% vs. 3.5%; p < 0.001). Children aged 5–14 years had the highest age-specific prevalence (31.3%; 95% CI: 22.4–41.9%) and accounted for 38.8% of cases. In multivariable analysis, brucellosis was associated with age 5–14 years (aPR 2.35; 95% CI: 1.51–3.68), prolonged fever >7 days (aPR 1.83; 95% CI: 1.20–2.78), and muscle pain (aPR 2.29; 95% CI: 1.02–5.13). Under a restricted case definition, prevalence decreased to 11.6%, but core associations remained directionally consistent.

**Conclusions/Significance:**

Brucellosis was common among febrile patients at two Kenyan pastoral health facilities, with highest prevalence among children aged 5–14 years and marked site heterogeneity. Findings support improved clinical recognition of brucellosis in pastoral facilities and further investigation of age-patterned household exposure pathways.

## Author summary

Brucellosis is a bacterial infection spread from livestock to people, common in communities that depend on animals for their livelihood. In Kenya’s pastoral regions, families live in close contact with cattle, goats, sheep, and camels, regularly consuming fresh milk and assisting with animal births–activities that can transmit the disease. We tested 439 patients with fever at two health facilities in contrasting pastoral areas of Kenya and found that about 1 in 7 had brucellosis. The burden was not evenly distributed: one site had five times more cases than the other, likely reflecting differences in how livestock are managed and how much animals move and mix. Children aged 5–14 years had the highest prevalence – nearly one in three met the case definition – and represented about 40% of all cases. This age pattern may reflect children’s participation in animal care, herding, and consumption of unpasteurized dairy products in pastoral households, though the specific exposure pathways were not directly measured. Prolonged fever and muscle pain were associated with brucellosis in multivariable analysis, suggesting that these clinical features should raise suspicion for brucellosis among febrile patients in pastoral settings. A sensitivity analysis using more stringent laboratory criteria reduced the prevalence estimate but preserved the main epidemiologic patterns.

## Introduction

Brucellosis remains a leading zoonotic infection globally, with an estimated 2.1 million new human cases annually, though true incidence is substantially underreported in endemic regions [1]. Sub-Saharan Africa bears a disproportionate burden, with community-based seroprevalence ranging from 2% to 44% depending on population and setting [2–4]. Facility-based studies among febrile patients in East Africa have reported prevalence estimates of 5–13%, though estimates vary with diagnostic methods and population characteristics [5–7]. In pastoralist communities, transmission occurs through ingestion of unpasteurized dairy products, direct animal contact during milking or herding, and exposure to reproductive materials during birthing assistance or slaughter [8–10].

In Kenya’s arid and semi-arid lands–which comprise 80% of the national territory and support approximately 40% of the population–pastoralist communities maintain daily contact with multiple livestock species (cattle, goats, sheep, and camels) through herding, milking, animal husbandry, and reproductive management tasks [11]. This human–animal interface supports transmission through herd-level conditions that sustain infection in livestock–including shared water points, inter-herd mixing during seasonal migration, and limited veterinary services–as well as direct human exposures such as milking, handling birthing materials, and consumption of unpasteurized dairy products including camel milk [9,10]. Transmission risk varies with livestock management practices, herd mobility, species composition, and access to veterinary services across pastoral and agropastoral contexts. Despite endemic livestock brucellosis, systematic facility-based surveillance for human brucellosis in pastoral populations remains limited.

Clinical diagnosis of brucellosis is complicated by non-specific symptoms overlapping malaria and typhoid fever [12,13]. Accurate diagnosis requires multi-method approaches: the Rose Bengal Test (RBT) offers rapid screening but remains underutilized in sub-Saharan Africa; ELISA may improve specificity but depends on laboratory infrastructure; and real-time PCR provides highly specific detection of *Brucella* DNA but is constrained by laboratory capacity in resource-limited settings [12,14]. These constraints underscore the need for integrated diagnostic approaches adapted to surveillance in pastoral settings.

Surveillance for brucellosis in sub-Saharan Africa is hindered by limited capacity for gold-standard methods like bacterial culture or paired serology, which are rarely feasible in pastoral settings due to biosafety concerns and difficulties in follow-up among mobile populations [15,16]. As a result, prevalence estimates vary widely, even within similar populations, and facility-based studies often rely on single diagnostic methods that underestimate the true burden [5,14]. Prior studies have disproportionately focused on adults and older children presumed to have occupational exposure, overlooking household transmission pathways and age-specific vulnerabilities. Children’s roles in milk collection and animal care are rarely assessed, yet data from similar contexts suggest meaningful exposure risk [6,17]. Data from pastoral communities in Tanzania and Ethiopia suggest that children participate in animal care and milk handling from an early age, yet age-disaggregated brucellosis prevalence data from facility-based settings remain scarce [4,6,7].

To address these gaps, we conducted prospective facility-based surveillance at two ecologically distinct pastoral health facilities in Kenya, embedded within a broader One Health cohort investigating *Brucella* spp. transmission dynamics [18]. Our objectives were to: (1) estimate brucellosis prevalence among patients presenting with acute febrile illness at two ecologically distinct pastoral health facilities; (2) identify demographic, clinical, and exposure factors associated with brucellosis; and (3) describe geographic heterogeneity in disease burden between sites.

## Methods

### Ethics statement

Written informed consent was obtained from participants aged ≥18 years. For participants aged <18 years, written informed consent was obtained from a parent or guardian. Written assent was obtained from participants aged 13–17 years, as required under the approved protocol. The study was approved by the Kenya Medical Research Institute (KEMRI) Scientific and Ethics Review Unit (Protocol No. 4405), reliance obtained from Washington State University Institutional Review Board and authorized by the Kenya Ministry of Health and National Commission for Science, Technology and Innovation (License No: NACOSTI/P/22/17621).

### Study design and setting

**Fig 1:**
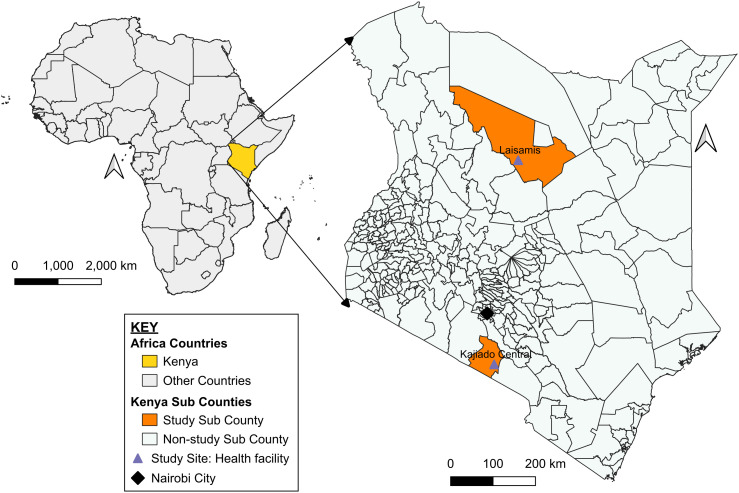
Map of the study sites. Orange areas indicate study subcounties; triangles show health facility locations; black diamond indicates capital city. Map generated in QGIS. Kenya administrative boundary shapefiles from the Humanitarian Data Exchange (https://data.humdata.org/dataset/cod-ab-ken), source IEBC, contributed by OCHA FISS, licensed under CC BY-IGO. Africa continent boundaries from Natural Earth (https://www.naturalearthdata.com), public domain.

From February 2023 to July 2024, we conducted prospective surveillance at two sentinel health facilities: Laisamis Sub-County Referral Hospital (Marsabit County) and Mailwa Health Center (Kajiado County) (Fig 1). These sites were purposively selected based on reported livestock brucellosis burden and distinct ecological characteristics. Laisamis serves a highly mobile pastoralist population managing mixed herds of camels, cattle, sheep and goats across vast, communal rangelands. In contrast, Mailwa serves agropastoral communities where land tenure changes have led to subdivision of traditional grazing areas and more restricted livestock mobility [19,20]. This ecological heterogeneity created natural variation in transmission-relevant factors–including herd mixing, animal density, and mobility–enabling exploration of whether geographic variation in human brucellosis burden reflects local livestock management and environmental conditions. The study represents the human surveillance arm of a broader One Health longitudinal cohort examining *Brucella spp.* transmission dynamics in pastoral Kenya [18]. The two facilities serve ecologically distinct pastoral and agropastoral populations. However, because only two facilities were included, observed site differences cannot be fully separated from unmeasured facility-, population-, and livestock-management factors.

### Study population

Eligible participants were aged ≥1 year and presented with acute febrile illness, defined as self-reported fever within the preceding 21 days or measured axillary temperature ≥38.0°C, accompanied by at least one brucellosis-compatible symptom: night sweats, joint pain, headache, fatigue, or anorexia. The 38.0°C axillary threshold follows WHO Integrated Disease Surveillance and Response guidelines for acute febrile illness investigation [16]. The 21-day symptom-history window was selected to capture the subacute presentation characteristic of human brucellosis [12]. We excluded individuals with documented bleeding disorders precluding safe phlebotomy or those requiring immediate clinical stabilization. Enrollment proceeded among all consecutive patients meeting criteria during facility operating hours (Monday–Friday, 08:00–17:00).

### Data collection

Trained study personnel administered structured questionnaires capturing demographic characteristics (age, sex, education, occupation, household composition, livestock holdings), clinical presentation (fever duration, symptom progression, prior treatments), and exposure history during the three months preceding illness onset. Exposure assessment focused on established transmission pathways: consumption of unpasteurized dairy products by animal species, direct contact with livestock (feeding, milking, herding), and participation in high-risk reproductive practices (assisting births, handling placental and fetal materials, managing abortion events). Species-specific frequencies and behaviors were recorded to enable construction of composite exposure variables reflecting raw milk consumption intensity (categorized as low/none, moderate, high) and animal contact diversity (≤1, 2, ≥ 3 species). All data were electronically captured in REDCap [21] with real-time validation, and field supervisors conducted regular audits with discrepancy resolution through source document review and participant re-contact.

### Laboratory procedures

Venous blood samples (2–5 mL) were collected and transported at 2–8°C in insulated cold boxes with ice packs to the field laboratory for serum separation, then shipped to Kenya Medical Research Institute Centre for Virus Research (KEMRI-CVR) for storage at −80°C until analysis. Rose Bengal Test (RBT) was performed using a semiquantitative serial dilution protocol [14], in which sera were tested at two-fold dilutions (neat through 1:32) against Rose Bengal antigen (Pourquier, IDEXX). This approach, which extends the standard qualitative RBT to provide titer information, has been validated in East African pastoral populations [14,22]. Titers ≥1:8 were considered indicative of infection, in line with prior findings including from East Africa [22]. Real-time PCR was performed using assays targeting the *Brucella* spp. IS*711* and *bcsp*31 genomic regions, respectively [23,24]. Samples were considered PCR positive when amplification was observed in both assay targets. IgG antibodies were measured using a commercial indirect ELISA (Brucella IgG ELISA, IBL, Minneapolis, MN, USA) per manufacturer protocol, with classification based on lot-specific OD cutoffs.

### Brucellosis case classification

All enrolled participants met the acute febrile illness eligibility criterion and reported at least one brucellosis-compatible symptom, as defined above. Within this clinically eligible population, brucellosis cases were identified using a hierarchical, mutually exclusive analytic classification based on laboratory results:

(1) RBT titer ≥1:8;
(2) PCR detection of *Brucella* DNA with low-titer RBT reactivity (excluding participants meeting criterion 1); or
(3) Low-titer RBT reactivity with ELISA IgG positivity (excluding participants meeting criteria 1 or 2).

Low-titer RBT reactivity was defined as visible agglutination at 1:2 or 1:4 dilution but negative at ≥1:8. Participants meeting any criterion were classified as cases; participants with complete laboratory results meeting none were classified as non-cases.

The requirement for RBT reactivity as a gating criterion for Tier 3 classification reflects evidence that RBT demonstrates sensitivity of 87.5–100% for detecting acute brucellosis in studies using appropriate comparison groups [25]. Low-titer RBT reactivity, while below the ≥ 1:8 threshold used for Tier 1, nonetheless indicates detectable agglutinating antibody whose diagnostic significance is strengthened when corroborated by a second serological method. ELISA IgG positivity without any RBT reactivity was not classified as a case, as isolated ELISA positivity may reflect cross-reactive antibodies or past resolved infection rather than current brucellosis [14,25]. This analytic classification was developed for epidemiologic inference in a facility-based surveillance context where culture and paired serology were not feasible. It differs from WHO confirmed case criteria, which require bacteriological confirmation or seroconversion. A sensitivity analysis restricting case classification to RBT ≥ 1:8 or PCR positivity was conducted to assess robustness to classification choice. Participants were classified as cases or non-cases only when sufficient laboratory information was available to apply the case definition. Enrolled participants lacking sufficient laboratory information for final classification were excluded from the primary analytic dataset.

### Statistical analysis

The study design, surveillance procedures, and laboratory methods were specified in the study protocol and have been described in detail [18]. Because bacterial culture and paired serology were not feasible in this surveillance context, an analytic case classification integrating RBT, PCR, and ELISA results was applied for epidemiologic analysis. Sensitivity analyses restricting the definition to RBT ≥ 1:8 or PCR positivity were conducted to evaluate the robustness of findings to case classification. Analyses were performed in R version 4.3.2 (R Core Team, 2023). Categorical variables are presented as frequencies and percentages; continuous variables as medians and interquartile ranges due to expected non-normality. Between-group comparisons employed chi-square or Fisher’s exact tests (when expected counts <5) for categorical variables, and Mann-Whitney U tests for continuous variables. Missingness across analytic variables was minimal and analyses were conducted using complete-case observations.

Brucellosis prevalence was estimated as the proportion of cases meeting laboratory criteria, with 95% confidence intervals calculated using the Wilson score method. Site-specific and age-stratified prevalence estimates were compared using two-proportion z-tests. Bivariate associations between case status and demographic, clinical, or exposure variables were examined using chi-square, Fisher’s exact, or Mann-Whitney U tests as appropriate. Composite exposure variables were constructed as follows: raw milk consumption intensity (low/none, moderate [single species or infrequent], or high [multiple species with frequent unpasteurized consumption]); animal contact diversity (low [≤1 species], moderate [2 species], or high [≥3 species]); and reproductive exposure risk (low, moderate, or high based on frequency of assisting animal births and handling placental or fetal materials).

Given the outcome prevalence of 15.3%, modified Poisson regression with robust (Huber–White sandwich) variance estimation was used to estimate adjusted prevalence ratios and 95% confidence intervals [26]. Variables achieving p < 0.20 in bivariate analyses were considered candidates for multivariable modeling. From this candidate set, the final clinical and exposure covariates were selected through backward elimination guided by Akaike Information Criterion, retaining age group, fever duration, muscle pain, joint pain, and sheep contact in the past 3 months. Sex was retained as a demographic confounder, and study site was included because of the substantial difference in prevalence between sites. Age was categorized as 1– < 5, 5–14, 15–49, and ≥50 years. Multicollinearity was assessed using variance inflation factors; overdispersion was evaluated using deviance-to-degrees-of-freedom ratios. Statistical significance was assessed at α = 0.05 (two-sided).

In sensitivity analysis, a restricted case definition requiring RBT titer ≥1:8 or PCR positivity was applied. Participants classified as cases only through low-titer RBT reactivity with ELISA IgG positivity under the primary definition were reclassified as non-cases. The analytic denominator was retained at 439 participants to evaluate the effect of case-definition specificity rather than define a different analytic population. Bivariate and multivariable analyses were repeated using the same covariate specification. Associations were considered stable if they maintained consistent direction and overlapping 95% confidence intervals across both definitions.

## Results

### Participant enrolment and study population

**Fig 2:**
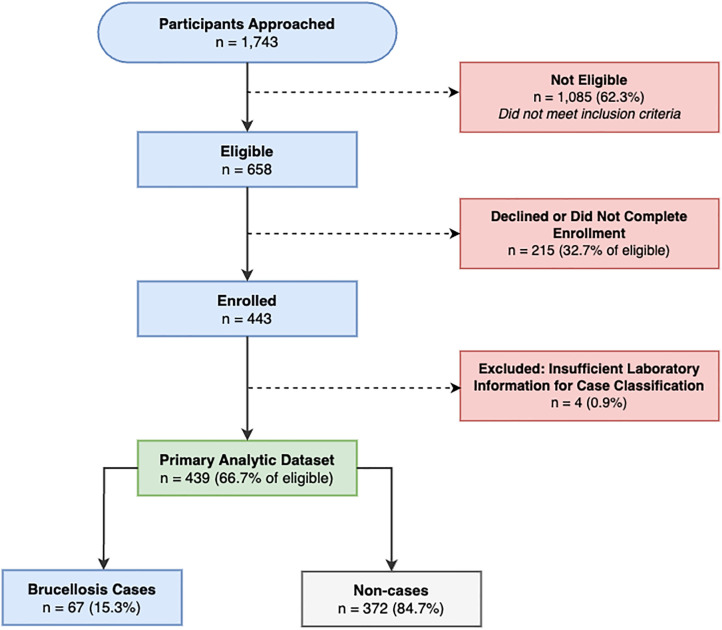
Participant enrolment and exclusion flowchart for brucellosis surveillance study across two pastoral sites in Kenya, February 2023-July 2024. The flow diagram summarizes screening, eligibility assessment, enrolment, laboratory exclusions, and inclusion in the primary analytic dataset.

Of 1,743 individuals with acute febrile illness screened, 1,085 (62.3%) did not meet eligibility criteria. Of 658 eligible participants, 215 (32.7%) were not enrolled. Enrolment was conducted during routine facility operating hours (Monday–Friday, 08:00–17:00). Non-enrolment primarily reflected operational constraints such as limited staffing capacity and participant time limitations rather than systematic exclusion based on clinical characteristics. Of 443 enrolled participants, four lacked sufficient laboratory information for final case classification and were excluded, yielding a primary analytic dataset of 439 participants (439/658 = 66.7% of eligible individuals). Participant flow is presented in Fig 2.

### Brucellosis prevalence

**Fig 3:**
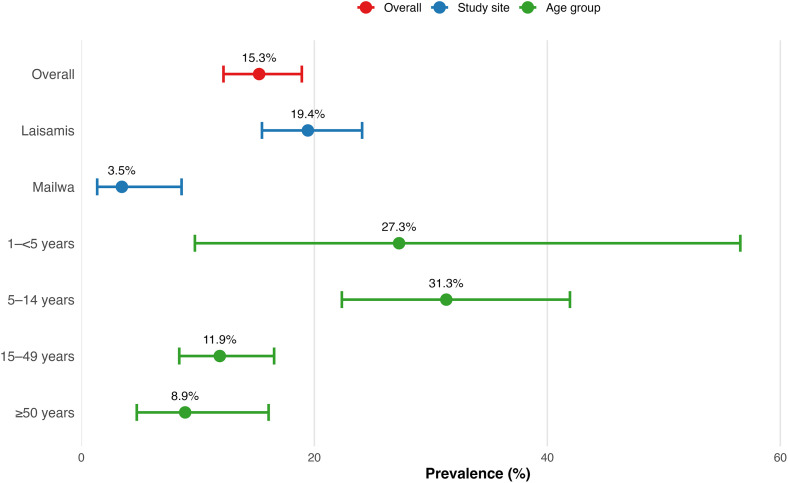
Brucellosis prevalence with 95% confidence intervals by study site and age group among febrile patients aged ≥1 year at two pastoral health facilities in Kenya.

Among 439 participants, 67 (15.3%; 95% CI: 12.1%–18.8%) met brucellosis case criteria (Fig 3). The hierarchical classification identified 26 cases (38.8%) via RBT titer ≥1:8 (Tier 1), 25 (37.3%) via PCR positivity with low-titer RBT reactivity (Tier 2), and 16 (23.9%) via low-titer RBT reactivity with ELISA IgG positivity (Tier 3). The detailed diagnostic patterns are presented in S1 Table.

Geographic variation was substantial: Laisamis reported 19.4% prevalence (95% CI: 15.5%–24.1%) compared to 3.5% in Mailwa (95% CI: 1.4%–8.6%; p < 0.001), representing a 5.5-fold difference between sites (Fig 3). Age-stratified prevalence estimates revealed marked variation across age groups. Children aged 5–14 years had the highest prevalence at 31.3% (95% CI: 22.4–41.9%), followed by children aged 1– < 5 years at 27.3% (95% CI: 9.7–56.6%). Adults aged 15–49 years had 11.9% prevalence, and adults aged ≥50 years 8.9%.

### Sociodemographic, clinical, and exposure characteristics

Cases were substantially younger than non-cases: median age 18.0 years (IQR: 11.0–34.7) versus 34.0 years (IQR: 20.0–49.0; p < 0.001). Children aged 5–14 years represented 38.8% of cases but only 18.9% of the analytic sample. Males comprised 59.7% of cases compared with 39.8% of non-cases (p = 0.004). Geographically, 94.0% of cases occurred at Laisamis. Bivariate comparisons of demographic, clinical, and exposure characteristics are presented in Table 1.

**Table 1:** Sociodemographic, clinical, and exposure characteristics by brucellosis case status among febrile participants at two pastoral health facilities in Kenya (N = 439).

Characteristic	Overall (N = 439)	Cases (N = 67)	Non-cases (N = 372)	P-value	Unadjusted PR (95% CI)
**Age (years), median (IQR)**	32.0 (17.0–48.0)	18.0 (11.0–34.7)	34.0 (20.0–49.1)	<0.001	—
**Age group**				<0.001	
1– < 5 years	11 (2.5)	3 (4.5)	8 (2.2)		2.21 (1.02–4.79)
5–14 years	83 (18.9)	26 (38.8)	57 (15.3)		2.35 (1.50–3.70)
15–49 years	244 (55.6)	29 (43.3)	215 (57.8)		Reference
≥50 years	101 (23.0)	9 (13.4)	92 (24.7)		0.68 (0.33–1.37)
**Sex**				0.004	
Female	251 (57.2)	27 (40.3)	224 (60.2)		Reference
Male	188 (42.8)	40 (59.7)	148 (39.8)		1.98 (1.26–3.10)
**Study site**				<0.001	
Mailwa	115 (26.2)	4 (6.0)	111 (29.8)		Reference
Laisamis	324 (73.8)	63 (94.0)	261 (70.2)		5.59 (2.08–15.01)
**Education level**				0.018	
No formal education	325 (74.0)	52 (77.6)	273 (73.4)		Reference
Child	14 (3.2)	6 (9.0)	8 (2.2)		2.68 (1.36–5.27)
Primary	52 (11.8)	4 (6.0)	48 (12.9)		0.48 (0.18–1.27)
Secondary and above	48 (10.9)	5 (7.5)	43 (11.6)		0.65 (0.28–1.53)
**Occupation**				<0.001	
Non-herder	113 (25.7)	10 (14.9)	103 (27.7)		Reference
Herder	107 (24.4)	18 (26.9)	89 (23.9)		1.90 (0.92–3.93)
Housewife/Houseworker	144 (32.8)	15 (22.4)	129 (34.7)		1.18 (0.55–2.51)
Minor (<12 years)	75 (17.1)	24 (35.8)	51 (13.7)		3.62 (1.83–7.14)
**Fever duration**				<0.001	
≤7 days	344 (78.4)	38 (56.7)	306 (82.3)		Reference
>7 days	95 (21.6)	29 (43.3)	66 (17.7)		2.76 (1.80–4.23)
**Constitutional symptoms**					
Joint pain	356 (81.1)	66 (98.5)	290 (78.0)	<0.001	15.39 (2.17–109.27)
Muscle pain	304 (69.2)	61 (91.0)	243 (65.3)	<0.001	4.51 (2.00–10.19)
Fatigue	369 (84.1)	65 (97.0)	304 (81.7)	0.003	6.17 (1.55–24.60)
Chills	175 (39.9)	42 (62.7)	133 (35.8)	<0.001	2.53 (1.61–4.00)
Night sweats	134 (30.5)	29 (43.3)	105 (28.2)	0.020	1.74 (1.12–2.69)
Back pain	317 (72.2)	59 (88.1)	258 (69.4)	0.003	2.84 (1.40–5.76)
Headache	390 (88.8)	63 (94.0)	327 (87.9)	0.209	1.98 (0.75–5.20)
**Other symptoms**					
Cough	103 (23.5)	25 (37.3)	78 (21.0)	0.006	1.94 (1.25–3.02)
Loss of appetite	80 (18.2)	11 (16.4)	69 (18.5)	0.807	0.88 (0.48–1.60)
Sore throat	36 (8.2)	4 (6.0)	32 (8.6)	0.631	0.71 (0.27–1.84)
**Any chronic condition**	41 (9.3)	4 (6.0)	37 (9.9)	0.423	0.62 (0.24–1.61)
**Raw milk consumption intensity**				<0.001	
Low/No intensity	19 (4.3)	1 (1.5)	18 (4.8)		Reference
Moderate intensity (1 species)	79 (18.0)	3 (4.5)	76 (20.4)		0.72 (0.08–6.64)
High intensity (≥2 species)	341 (77.7)	63 (94.0)	278 (74.7)		3.51 (0.51–24.22)
**Animal contact diversity**				0.133	
Low diversity	35 (8.0)	4 (6.0)	31 (8.3)		Reference
Moderate diversity	95 (21.6)	9 (13.4)	86 (23.1)		0.83 (0.27–2.54)
High diversity	309 (70.4)	54 (80.6)	255 (68.5)		1.53 (0.59–3.97)
**Species-specific contact (past 3 months)**					
Sheep contact	398 (90.7)	66 (98.5)	332 (89.2)	0.030	6.80 (0.97–47.71)
Cattle contact	174 (39.6)	20 (29.9)	154 (41.4)	0.100	0.65 (0.40–1.05)
**Reproductive exposure intensity**				0.056	
Low/No exposure	367 (83.6)	50 (74.6)	317 (85.2)		Reference
Moderate exposure	2 (0.5)	1 (1.5)	1 (0.3)		3.67 (0.76–17.72)
High exposure	70 (15.9)	16 (23.9)	54 (14.5)		1.68 (1.00–2.81)
**Occupational exposures**					
Butchering	134 (30.5)	30 (44.8)	104 (28.0)	0.009	1.85 (1.19–2.86)
Handling hides/skins	284 (64.7)	49 (73.1)	235 (63.2)	0.152	1.49 (0.90–2.46)
Livestock stillbirths (past 6 months)	247 (56.3)	43 (64.2)	204 (54.8)	0.199	1.39 (0.88–2.21)
**Any milk consumption**	423 (96.4)	66 (98.5)	357 (96.0)	0.505	2.50 (0.37–16.87)

Data are n (%) unless otherwise stated. Percentages in Cases and Non-cases columns represent row percentages. Unadjusted prevalence ratios and 95% confidence intervals are shown where applicable. P-values from Pearson’s chi-square test, Fisher’s exact test (education level, age group), or Wilcoxon rank-sum test (age). Reference categories are indicated for categorical variables.

Cases presented with different clinical profiles from non-cases. Prolonged fever (>7 days) was twice as common in cases (43.3% vs. 17.7%; p < 0.001), while constitutional symptoms were near-universal among cases: joint pain (98.5% vs. 78.0%; p < 0.001), muscle pain (91.0% vs. 65.3%; p < 0.001), fatigue (97.0% vs. 81.7%; p = 0.003), and chills (62.7% vs. 35.8%; p < 0.001). In addition, respiratory and musculoskeletal manifestations–back pain (88.1% vs. 69.4%; p = 0.002), cough (37.3% vs. 21.0%; p = 0.007)–were more frequent in cases (Table 1).

Exposure patterns differed between cases and non-cases. Raw milk consumption was almost universal in both groups (98.5% and 96.0%, respectively), but consumption intensity was different: 94.0% of cases reported high-intensity consumption (multiple livestock species) versus 74.9% of non-cases (p < 0.001). Contact with small ruminants was also nearly ubiquitous in cases: 98.5% reported recent sheep contact compared to 89.2% of non-cases (p = 0.030). Butchering was reported by 44.8% of cases compared with 28.0% of non-cases (p = 0.009).

### Multivariable analysis

**Fig 4:**
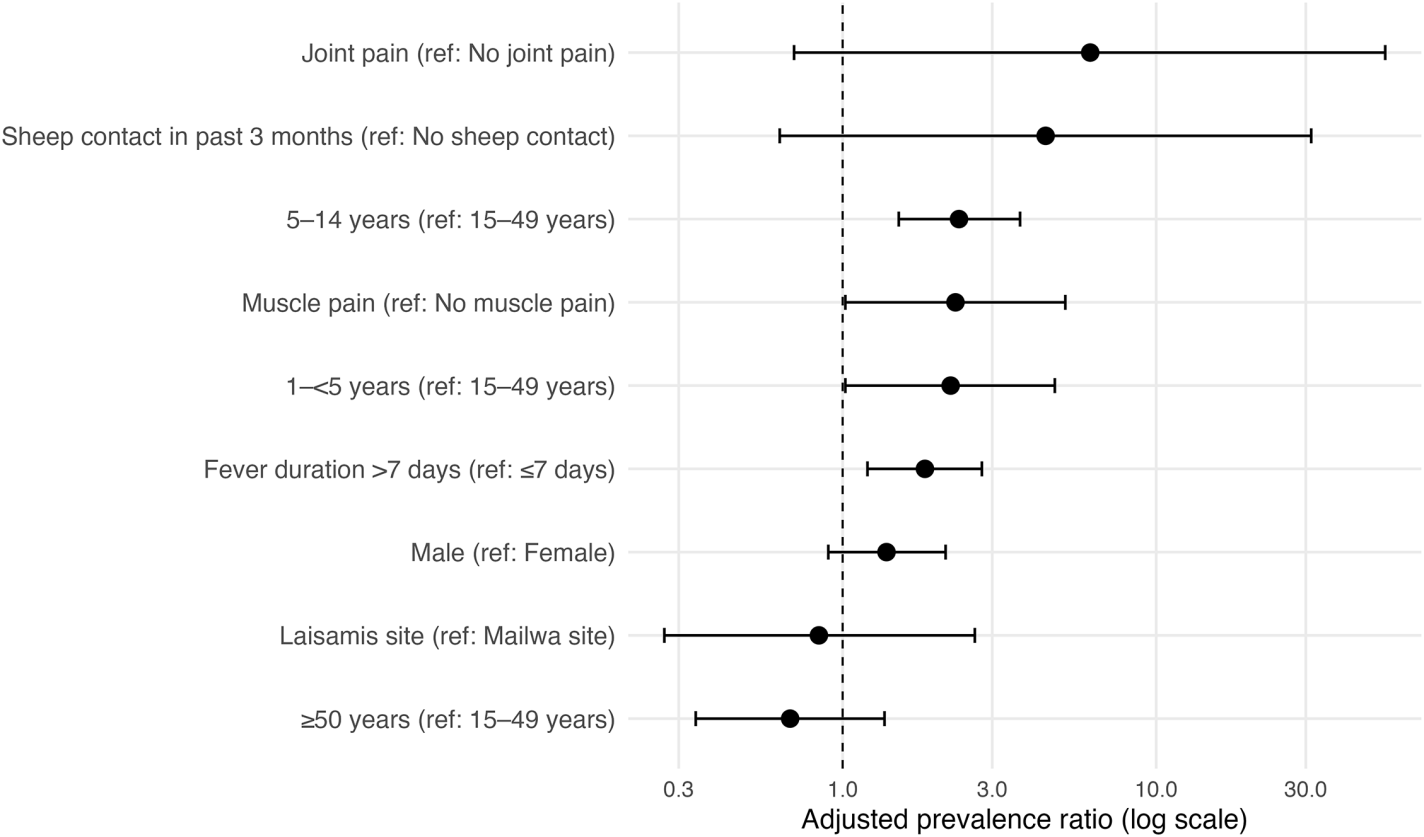
Adjusted prevalence ratios and 95% confidence intervals for factors associated with brucellosis among febrile patients at two pastoral health facilities in Kenya. Estimates from modified Poisson regression with robust variance estimation.

In multivariable analysis (Fig 4), children aged 5–14 years were more likely to meet the brucellosis case definition than adults aged 15–49 years (aPR 2.35; 95% CI: 1.51–3.68; p < 0.001). Prolonged fever >7 days (aPR 1.83; 95% CI: 1.20–2.78; p = 0.005) and muscle pain (aPR 2.29; 95% CI: 1.02–5.13; p = 0.043) were also associated with brucellosis. Children aged 1– < 5 years had an increased prevalence ratio (aPR 2.21; 95% CI: 1.02–4.75; p = 0.043) but this subgroup was small (n = 11). Joint pain and sheep contact in the past 3 months had increased point estimates but wide confidence intervals and were not statistically significant after adjustment. Study site was retained in the model but the adjusted site coefficient was not significant (aPR 0.84; 95% CI: 0.27–2.64; p = 0.762).

### Sensitivity analysis

Under the restricted case definition, 16 Tier 3 cases were reclassified as non-cases while retaining the denominator of 439 participants. Prevalence decreased from 15.3% (95% CI: 12.2–18.9%) to 11.6% (95% CI: 8.9–15.0%). Laisamis prevalence declined from 19.4% to 14.5% (all 16 borderline cases occurred at this site), while Mailwa remained unchanged at 3.5%. The distribution of reclassified cases across strata is presented in S2 Table.

Despite this diagnostic restriction, core associations remained robust and directionally consistent (S3 Table). Children aged 5–14 years retained the strongest association (aPR 2.90; 95% CI: 1.69–4.97; p < 0.001). Prolonged fever (aPR 2.19; 95% CI: 1.33–3.62; p = 0.002) and children aged 1– < 5 years (aPR 3.15; 95% CI: 1.42–6.98; p = 0.005) also remained associated with brucellosis. Muscle pain was directionally consistent but did not reach statistical significance under the restricted definition (aPR 2.22; 95% CI: 0.87–5.63; p = 0.095), likely reflecting reduced precision with fewer cases rather than absence of association.

## Discussion

This facility-based surveillance study found a prevalence of 15.3% among febrile patients attending two pastoral health facilities in Kenya, with the highest age-specific prevalence among children aged 5–14 years. This prevalence exceeds most prior facility-based estimates in East Africa (5–13%) [5–7]. The higher prevalence likely reflects two complementary factors: the integrated diagnostic approach (RBT, PCR, ELISA) combined serologic and molecular tests to detect both acute and chronic infections; and persistent transmission-enabling conditions–limited livestock vaccination, minimal milk pasteurization, and reliance on fresh dairy products for household nutrition [14,27].

Children aged 5–14 years had the highest prevalence (31.3%) and represented 38.8% of all cases. While similar patterns have been reported in neighboring Tanzania [6], this contrasts with most East African studies emphasizing adult occupational risk [5,28]. Several factors may contribute. Cultural and dietary practices often prioritize unpasteurized milk for children, and children frequently participate in animal husbandry activities such as feeding, watering, and caring for small ruminants. Further studies that directly measure children’s milk consumption patterns and livestock-related activities in pastoral households are needed to clarify the mechanisms underlying this age pattern.

The five-fold geographic difference in prevalence (19.4% Laisamis vs. 3.5% Mailwa) reflects distinct ecological systems. Laisamis, characterized by extensive pastoral mobility and seasonal herd migration, may facilitate more frequent inter-herd contact and *Brucella* spp. transmission. Mailwa has undergone land tenure restructuring with grazing subdivision and settlement, which may constrain livestock movement. This ecological distinction aligns with spatial mapping of brucellosis risk in Kenya, where mobile pastoralism is associated with higher livestock seroprevalence [3,19]. The geographic variation indicates that human brucellosis burden in pastoral health facilities is context-specific rather than uniform across pastoral settings, though the two-site design limits the ability to fully disentangle site-specific factors from broader ecological mechanisms. Site was retained as a covariate in the multivariable model to account for this heterogeneity. The adjusted site coefficient was not statistically significant (aPR 0.84; 95% CI: 0.27–2.64), but this does not negate the descriptive concentration of cases in Laisamis; rather, it indicates that individual-level covariates in the model partially account for the between-site difference.

Prolonged fever (>7 days) was associated with brucellosis in both primary (aPR 1.83; p = 0.005) and sensitivity (aPR 2.19; p = 0.002) analyses. This aligns with brucellosis’s classically subacute course, distinguishing it from acute infections like malaria or pneumonia that typically resolve within a few days [29]. In resource-limited pastoralist settings where laboratory confirmation may be delayed, prolonged fever–especially when accompanied by musculoskeletal symptoms–should raise clinical suspicion for brucellosis. Cough was also more frequently reported among cases (37.3% vs. 21.0%; p = 0.007), but given its non-specificity in outpatient febrile illness, this finding should be interpreted cautiously. The association may reflect co-infections or symptom clustering rather than a diagnostically discriminating feature.

Muscle pain was more common among cases (91.0% vs. 65.3%) and associated with brucellosis in the primary analysis (aPR 2.29; 95% CI: 1.02–5.13; p = 0.043). This association remained directionally consistent but did not reach statistical significance in the restricted-definition sensitivity analysis (aPR 2.22; 95% CI: 0.87–5.63; p = 0.095), likely reflecting reduced precision with fewer cases rather than absence of association. Prolonged fever, by contrast, retained predictive value across models, reinforcing its utility as a consistent clinical signal. The co-occurrence of prolonged fever musculoskeletal pain may help frontline clinicians differentiate brucellosis from other febrile illnesses in endemic pastoral areas.

Raw milk consumption and direct animal contact were not retained in the final multivariable model or were not statistically significant after adjustment. Several mechanisms may explain this finding. First, these exposures are near-universal in this population (96.8% reported any milk consumption; 90.7% reported sheep contact), limiting individual-level variation in adjusted models. Second, the high prevalence of these exposures across the study population limits their ability to discriminate individual-level risk in multivariable models. Third, collinearity or confounding by age, sex, or clinical presentation may have attenuated their independent effects. The factors associated with brucellosis in adjusted analysis were age 5–14 years, prolonged fever, and muscle pain, suggesting that demographic and clinical features discriminated cases more effectively than reported exposure history in this facility-based population. Given strong site clustering of cases (94% from Laisamis), some exposure associations–particularly raw milk consumption intensity–may partially reflect site-level ecology and pastoral mobility patterns rather than individual-level causal effects. The multivariable model adjusted for site, but complete disentanglement of individual and ecological effects was not possible given the observed distribution.

Species-specific livestock exposure variables require cautious interpretation. Sheep and goats are often co-managed in pastoral systems, and participants may not distinguish between species-specific contacts with precision, introducing potential exposure misclassification. However, human exposure pathways may differ by species and activity even where animals share pasture: goat exposure may occur primarily through milk consumption, whereas sheep contact may more often reflect direct handling or reproductive activities. In this study, goat-related exposure variables (handling, milk consumption, assisting births, placenta contact) were assessed; goat milk consumption was associated with case status in bivariate analysis, but direct goat handling and goat reproductive exposures were not. Sheep contact had an elevated point estimate but was imprecise after adjustment and should be interpreted as exploratory rather than evidence of a sheep-specific causal pathway.

Sixteen cases (23.9%) were classified through low-titer RBT reactivity and ELISA IgG positivity (Tier 3). The restricted case-definition sensitivity analysis reduced the prevalence estimate from 15.3% to 11.6%, indicating that the broader primary definition increased case ascertainment through inclusion of this tier. The principal age and clinical patterns remained directionally consistent under the restricted definition, supporting the robustness of the main findings while acknowledging uncertainty in the absolute prevalence estimate attributable to case-definition choice.

If the higher prevalence among children aged 5–14 years reflects household-level exposure pathways, prevention approaches for this age group may need to address household milk handling and children’s livestock contact rather than occupational risk alone. In high-prevalence pastoral settings, prolonged fever accompanied by musculoskeletal symptoms should raise clinical suspicion for brucellosis, particularly in children. The diagnostic approach combining RBT, PCR, and ELISA demonstrated operational feasibility; facility-level capacity for RBT screening with referral pathways for confirmatory testing warrants consideration as a practical implementation pathway. Coordination between clinical and veterinary services, informed by shared laboratory results, enables linked surveillance across human and animal health sectors.

Several limitations warrant acknowledgment. The cross-sectional design precludes causal inference; prospective follow-up from the nested longitudinal cohort [18] will help establish temporality. As a facility-based study among febrile care-seekers, the prevalence estimates should not be interpreted as community-level burden. Second, most cases occurred in Laisamis, and Mailwa contributed only four cases. The pooled risk factor analysis is therefore predominantly driven by the Laisamis population, and site-stratified multivariable analysis was not performed because of insufficient cases at Mailwa. Third, approximately one-third of eligible patients were not enrolled, and demographic or exposure information was not collected from non-enrolled individuals. If non-enrollees differed from enrollees in age distribution, mobility, or livestock-related exposures – for example, if mobile adult herders were less available during facility hours – the enrolled population may not fully represent the eligible population. Fourth, species-specific livestock exposures may be subject to misclassification because sheep and goats are often co-managed in pastoral systems, and some adjusted exposure estimates were imprecise. Fifth, the primary case definition included participants with low-titer RBT and ELISA IgG positivity; sensitivity analysis excluding these participants reduced the prevalence estimate but preserved the main associations. Finally, findings are derived from two ecologically distinct pastoral sites and may not generalize to other pastoral or agropastoral contexts.

This study found a brucellosis prevalence of 15.3% among febrile patients at two pastoral health facilities in Kenya, with marked geographic variation consistent with differences in livestock management between sites. Children aged 5–14 years had the highest age-specific prevalence. These findings support improved clinical recognition of brucellosis among febrile patients in pastoral facilities and further investigation of the exposure pathways underlying the age-specific burden pattern, within the framework of Kenya’s national brucellosis control strategy [30].

## Supporting information

S1 Table Brucellosis case classification by diagnostic criteria and study site (N = 439), February 2023–July 2024. (DOCX)

S2 Table Brucellosis prevalence under primary and restricted case definitions by study stratum. (DOCX)

S3 Table Sensitivity of adjusted prevalence ratios for factors associated with brucellosis under primary and restricted case definitions. (DOCX)

S1 Checklist STROBE Statement—Checklist of items that should be included in reports of cross-sectional studies. The STROBE checklist is reproduced under a Creative Commons Attribution 4.0 International License (CC BY 4.0); the original is available at https://www.strobe-statement.org/.
(DOCX)

## References

[1] Laine CG, Johnson VE, Scott HM, Arenas-Gamboa AM. Global estimate of human brucellosis incidence. Emerg Infect Dis. 2023;29(9):1789–97. doi: 10.3201/eid2909.230052 37610167 PMC10461652

[2] Njeru J, Melzer F, Wareth G, El-Adawy H, Henning K, Pletz MW. Human brucellosis in febrile patients seeking treatment at remote hospitals, northeastern Kenya, 2014–2015. Emerg Infect Dis. 2016;22:2160. doi: 10.3201/EID2212.160285 27662463 PMC5189133

[3] Osoro EM, Munyua P, Omulo S, Ogola E, Ade F, Mbatha P, et al. Strong Association between human and animal brucella seropositivity in a linked study in Kenya, 2012-2013. Am J Trop Med Hyg. 2015;93(2):224–31. doi: 10.4269/ajtmh.15-0113 26101275 PMC4530738

[4] Asrie F, Birhan M, Dagnew M, Berhane N. Prevalence of human brucellosis in Ethiopia: systematic review and meta-analysis. BMC Infect Dis. 2025;25(1):365. doi: 10.1186/s12879-025-10502-8 40089675 PMC11910861

[5] Njeru J, Wareth G, Melzer F, Henning K, Pletz MW, Heller R, et al. Systematic review of brucellosis in Kenya: disease frequency in humans and animals and risk factors for human infection. BMC Public Health. 2016;16(1):853. doi: 10.1186/s12889-016-3532-9 27549329 PMC4994226

[6] Bodenham RF, Lukambagire AS, Ashford RT, Buza JJ, Cash-Goldwasser S, Crump JA, et al. Prevalence and speciation of brucellosis in febrile patients from a pastoralist community of Tanzania. Sci Rep. 2020;10(1):7081. doi: 10.1038/s41598-020-62849-4 32341414 PMC7184621

[7] Sileshi B, Gizaw S, Merkeb B, Bekele T, Tadesse W, Kezali J, et al. Sero-prevalence of human brucellosis and associated factors among febrile patients attending Moyale Primary Hospital, Southern Ethiopia, 2023: evidences from pastoralist community. PLoS Negl Trop Dis. 2024;18:e0012715. doi: 10.1371/JOURNAL.PNTD.0012715 PMC11651574 39689060

[8] Mcdermott JJ, Grace D, Zinsstag J. Economics of brucellosis impact and control in low-income countries. Rev Sci Tech. 2013. doi: 10.20506/rst.32.1.2197 23837382

[9] McDermott JJ, Arimi SM. Brucellosis in sub-Saharan Africa: epidemiology, control and impact. Vet Microbiol. 2002;90(1–4):111–34. doi: 10.1016/s0378-1135(02)00249-3 12414138

[10] Corbel MMJ. Brucellosis in humans and animals. Geneva, Switzerland: World Health Organization, Food and Agriculture Organization of the United Nations, World Organization for Animal Health; 2006. doi: 10.2105/AJPH.30.3.299

[11] Kenya National Bureau of Statistics. 2019 Kenya Population and Housing Census. Nairobi: Kenya National Bureau of Statistics; 2019. Available from: https://www.knbs.or.ke/?p=5621

[12] Dean AS, Crump L, Greter H, Hattendorf J, Schelling E, Zinsstag J. Clinical manifestations of human brucellosis: a systematic review and meta-analysis. PLoS Negl Trop Dis. 2012;6(12):e1929. doi: 10.1371/journal.pntd.0001929 23236528 PMC3516581

[13] Köse Ş, Serin Senger S, Akkoçlu G, Kuzucu L, Ulu Y, Ersan G, et al. Clinical manifestations, complications, and treatment of brucellosis: evaluation of 72 cases. Turk J Med Sci. 2014;44(2):220–3. doi: 10.3906/sag-1112-34 25536728

[14] Díaz R, Casanova A, Ariza J, Moriyón I. The Rose Bengal Test in human brucellosis: a neglected test for the diagnosis of a neglected disease. PLoS Negl Trop Dis. 2011;5(4):e950. doi: 10.1371/journal.pntd.0000950 21526218 PMC3079581

[15] Franc KA, Krecek RC, Häsler BN, Arenas-Gamboa AM. Brucellosis remains a neglected disease in the developing world: a call for interdisciplinary action. BMC Public Health. 2018;18(1):125. doi: 10.1186/s12889-017-5016-y 29325516 PMC5765637

[16] WHO Regional Office for Africa. Technical Guidelines for Integrated Disease Surveillance and Response in the African Region. 3rd ed. WHO | Regional Office for Africa; 2019 [cited 2025 Oct 19]. Available from: https://www.afro.who.int/publications/technical-guidelines-integrated-disease-surveillance-and-response-african-region-third

[17] Bett B, Jost C, Allport R, Mariner J. Using participatory epidemiological techniques to estimate the relative incidence and impact on livelihoods of livestock diseases amongst nomadic pastoralists in Turkana South District, Kenya. Prev Vet Med. 2009;90(3–4):194–203. doi: 10.1016/j.prevetmed.2009.05.001 19487039

[18] Oketch D, Njoroge R, Ngere I, Gachohi J, Waiguru S, Omia D, et al. Design of a prospective human-animal cohort study to evaluate the role of camels and other livestock species in the transmission of Brucella spp. to humans in Kenya. Int J Environ Res Public Health. 2025;22(12):1859. doi: 10.3390/ijerph22121859 41464492 PMC12733042

[19] Akoko JM, Mwatondo A, Muturi M, Wambua L, Abkallo HM, Nyamota R, et al. Mapping brucellosis risk in Kenya and its implications for control strategies in sub-Saharan Africa. Sci Rep. 2023;13(1):20192. doi: 10.1038/s41598-023-47628-1 37980384 PMC10657468

[20] Rotich SJ, Funder M, Marani M, Nathan I. Climate change adaptation and land tenure: exploring pastoral adaptation strategies under communal and private land ownership in Kenya. Local Environ. 2024;30(1):37–57. doi: 10.1080/13549839.2024.2428207

[21] Harris PA, Taylor R, Thielke R, Payne J, Gonzalez N, Conde JG. Research electronic data capture (REDCap)--a metadata-driven methodology and workflow process for providing translational research informatics support. J Biomed Inform. 2009;42(2):377–81. doi: 10.1016/j.jbi.2008.08.010 18929686 PMC2700030

[22] Lukambagire AS, Mendes ÂJ, Bodenham RF, McGiven JA, Mkenda NA, Mathew C, et al. Performance characteristics and costs of serological tests for brucellosis in a pastoralist community of northern Tanzania. Sci Rep. 2021;11(1):5480. doi: 10.1038/s41598-021-82906-w 33750848 PMC7943594

[23] Probert WS, Schrader KN, Khuong NY, Bystrom SL, Graves MH. Real-time multiplex PCR assay for detection of Brucella spp., B. abortus, and B. melitensis. J Clin Microbiol. 2004;42:1290–3. doi: 10.1128/JCM.42.3.1290-1293.2004 15004098 PMC356861

[24] Matero P, Hemmilä H, Tomaso H, Piiparinen H, Rantakokko-Jalava K, Nuotio L. Rapid field detection assays for Bacillus anthracis, Brucella spp., Francisella tularensis and Yersinia pestis. Clin Microbiol Infect. 2011;17:34–43. doi: 10.1111/j.1469-0691.2010.03178.x 20132255

[25] Ekiri AB, Kilonzo C, Bird BH, VanWormer E, Wolking DJ, Smith WA, et al. Utility of the rose bengal test as a point-of-care test for human brucellosis in endemic African settings: a systematic review. J Trop Med. 2020;2020:6586182. doi: 10.1155/2020/6586182 33014074 PMC7519193

[26] Zou G. A modified poisson regression approach to prospective studies with binary data. Am J Epidemiol. 2004;159:702–6. doi: 10.1093/AJE/KWH090 15033648

[27] Loubet P, Magnan C, Salipante F, Pastre T, Keriel A, O’Callaghan D, et al. Diagnosis of brucellosis: combining tests to improve performance. PLoS Negl Trop Dis. 2024;18(9):e0012442. doi: 10.1371/journal.pntd.0012442 39236075 PMC11407618

[28] Tumwine G, Matovu E, Kabasa JD, Owiny DO, Majalija S. Human brucellosis: sero-prevalence and associated risk factors in agro-pastoral communities of Kiboga District, Central Uganda. BMC Public Health. 2015;15:900. doi: 10.1186/s12889-015-2242-z 26374402 PMC4572625

[29] Dean AS, Crump L, Greter H, Schelling E, Zinsstag J. Global burden of human brucellosis: a systematic review of disease frequency. PLoS Negl Trop Dis. 2012;6(10):e1865. doi: 10.1371/journal.pntd.0001865 23145195 PMC3493380

[30] Zoonotic Disease Unit. National Strategy for the Prevention and Control of Brucellosis in Humans and Animals in Kenya (2021–2040). Nairobi; 2021.

