# Supplementary material for: High brucellosis prevalence and risk among children aged 5–14 years in Kenyan pastoral health facilities: A facility-based surveillance study
Source: PLoS Negl Trop Dis. 2026 Aug 10;20(8):e0014129. doi: 10.1371/journal.pntd.0014129 (PMC13472494; doi:10.1371/journal.pntd.0014129)
Supplement: Supplementary file 1 [file pntd.0014129.s001.docx]

*S1 Table. Brucellosis case classification by diagnostic criteria and study site (n=439), February 2023–July 2024.*

| **Classification tier** | **Definition** | **Total (n)** | **Laisamis (n)** | **Mailwa (n)** |
| --- | --- | --- | --- | --- |
| **Cases** |  |  |  |  |
| Tier 1: RBT titer ≥1:8 | RBT titer ≥1:8 | 26 | 26 | 0 |
| Tier 2: PCR positive | PCR positive with low-titer RBT reactivity (excluding Tier 1) | 25 | 21 | 4 |
| Tier 3: RBT + ELISA positive | Low-titer RBT reactivity with ELISA IgG positivity (excluding Tiers 1–2) | 16 | 16 | 0 |
| **Subtotal cases** |  | **67** | **63** | **4** |
| **Non-cases** |  |  |  |  |
| No laboratory evidence of brucellosis | Did not meet any case classification criterion | 372 | 261 | 111 |
| **Grand total** |  | **439** | **324** | **115** |
| Tiers are hierarchical and mutually exclusive: participants meeting a higher-tier criterion were not reclassified into lower tiers. Under the restricted case definition used in sensitivity analysis, Tier 3 cases were reclassified as non-cases (see Table S2). | | | | |
