# Supplementary material for: High brucellosis prevalence and risk among children aged 5–14 years in Kenyan pastoral health facilities: A facility-based surveillance study
Source: PLoS Negl Trop Dis. 2026 Aug 10;20(8):e0014129. doi: 10.1371/journal.pntd.0014129 (PMC13472494; doi:10.1371/journal.pntd.0014129)
Supplement: Supplementary file 2 [file pntd.0014129.s002.docx]

*S2 Table. Brucellosis prevalence under primary and restricted case definitions by study stratum.*

| **Stratum** | **Level** | **N** | **Primary definition: cases, prevalence (95% CI)** | **Restricted definition: cases, prevalence (95% CI)** | **Tier 3 reclassified** |
| --- | --- | --- | --- | --- | --- |
| Overall | Overall | 439 | 67 (15.3%; 95% CI: 12.2–18.9) | 51 (11.6%; 95% CI: 8.9–15.0) | 16 |
| Site | Laisamis | 324 | 63 (19.4%; 95% CI: 15.5–24.1) | 47 (14.5%; 95% CI: 11.1–18.8) | 16 |
| Site | Mailwa | 115 | 4 (3.5%; 95% CI: 1.4–8.6) | 4 (3.5%; 95% CI: 1.4–8.6) | 0 |
| Age group | 1–<5 years | 11 | 3 (27.3%; 95% CI: 9.7–56.6) | 3 (27.3%; 95% CI: 9.7–56.6) | 0 |
| Age group | 5–14 years | 83 | 26 (31.3%; 95% CI: 22.4–41.9) | 22 (26.5%; 95% CI: 18.2–36.9) | 4 |
| Age group | 15–49 years | 244 | 29 (11.9%; 95% CI: 8.4–16.5) | 20 (8.2%; 95% CI: 5.4–12.3) | 9 |
| Age group | ≥50 years | 101 | 9 (8.9%; 95% CI: 4.8–16.1) | 6 (5.9%; 95% CI: 2.8–12.4) | 3 |
| Primary definition: RBT titer ≥1:8, PCR positive, or low-titer RBT with ELISA IgG positivity. Restricted definition: RBT titer ≥1:8 or PCR positive only. Tier 3 cases were reclassified as non-cases under the restricted definition; denominators remain unchanged. 95% confidence intervals calculated using the Wilson score method. | | | | | |
