# Supplementary material for: High brucellosis prevalence and risk among children aged 5–14 years in Kenyan pastoral health facilities: A facility-based surveillance study
Source: PLoS Negl Trop Dis. 2026 Aug 10;20(8):e0014129. doi: 10.1371/journal.pntd.0014129 (PMC13472494; doi:10.1371/journal.pntd.0014129)
Supplement: Supplementary file 3 [file pntd.0014129.s003.docx]

***S3 Table. Sensitivity of adjusted prevalence ratios for factors associated with brucellosis under primary and restricted case definitions***

| **Variable** | **Reference** | **Primary aPR (95% CI)** | **Primary p-value** | **Restricted aPR (95% CI)** | **Restricted p-value** | **Sensitivity interpretation** |
| --- | --- | --- | --- | --- | --- | --- |
| 5–14 years | 15–49 years | 2.35 (1.50–3.70) | <0.001 | 2.90 (1.69–4.97) | <0.001 | Robust; significant under both definitions |
| 1–<5 years | 15–49 years | 2.21 (1.02–4.79) | 0.046 | 3.15 (1.42–6.98) | 0.005 | Robust; significant under both definitions |
| ≥50 years | 15–49 years | 0.68 (0.33–1.37) | 0.278 | 0.67 (0.28–1.62) | 0.374 | Directionally consistent; not statistically significant |
| Male | Female | 1.38 (0.89–2.14) | 0.148 | 1.54 (0.91–2.60) | 0.110 | Directionally consistent; not statistically significant |
| Laisamis | Mailwa | 0.84 (0.26–2.68) | 0.765 | 0.53 (0.16–1.80) | 0.312 | Directionally consistent; not statistically significant |
| Fever duration >7 days | ≤7 days | 1.83 (1.20–2.79) | 0.005 | 2.19 (1.33–3.62) | 0.002 | Robust; significant under both definitions |
| Muscle pain | No muscle pain | 2.29 (1.02–5.18) | 0.046 | 2.22 (0.87–5.63) | 0.095 | Directionally consistent; attenuated precision under restricted definition |
| Joint pain | No joint pain | 6.16 (0.69–55.12) | 0.104 | 6.46 (0.70–59.51) | 0.100 | Directionally consistent; imprecise |
| Sheep contact, past 3 months | No sheep contact | 4.44 (0.62–31.92) | 0.138 | 3.45 (0.46–25.68) | 0.227 | Directionally consistent; imprecise |
| aPR, adjusted prevalence ratio; CI, confidence interval. Sensitivity was assessed descriptively by comparing the direction, magnitude, precision, and statistical significance of adjusted prevalence ratios across the primary and restricted case definitions. “Robust” indicates that the direction of association and statistical significance were retained under both definitions. “Directionally consistent” indicates that the direction of association was retained, but estimates were imprecise or not statistically significant under one or both definitions. Confidence interval overlap was interpreted descriptively and was not treated as a formal statistical test of equality between estimates | | | | | | |
