# Supplementary material for: High brucellosis prevalence and risk among children aged 5–14 years in Kenyan pastoral health facilities: A facility-based surveillance study
Source: PLoS Negl Trop Dis. 2026 Aug 10;20(8):e0014129. doi: 10.1371/journal.pntd.0014129 (PMC13472494; doi:10.1371/journal.pntd.0014129)
Supplement: Supplementary file 4 [file pntd.0014129.s004.docx]

# **S4 Checklist. STROBE Statement—Checklist of items that should be included in reports of cross-sectional studies**

| **Section/Topic** | **Item #** | **Recommendation** | **Page/Section** | **Relevant Manuscript Text** |
| --- | --- | --- | --- | --- |
| Title and abstract | 1 | (a) Indicate the study's design with a commonly used term in the title or the abstract (b) Provide in the abstract an informative and balanced summary of what was done and what was found | *Title page; Abstract* | Title identifies the study as facility-based surveillance; abstract summarizes design, setting, methods, main findings, and sensitivity analysis. |
| **Introduction** |  |  |  |  |
| Background/rationale | 2 | Explain the scientific background and rationale for the investigation being reported | *Introduction* | Introduction describes brucellosis burden, diagnostic challenges, and evidence gaps in pastoral populations. |
| Objectives | 3 | State specific objectives, including any prespecified hypotheses | *Introduction, final paragraph* | Final Introduction paragraph states the three study objectives. |
| **Methods** |  |  |  |  |
| Study design | 4 | Present key elements of study design early in the paper | *Methods, Study Design and Setting* | Methods identify the study as prospective facility-based surveillance. |
| Setting | 5 | Describe the setting, locations, and relevant dates, including periods of recruitment, exposure, follow-up, and data collection | *Methods, Study Design and Setting* | Methods describe the two Kenyan pastoral health facilities, counties, study dates, and ecological context. |
| Participants | 6 | (a) Give the eligibility criteria, and the sources and methods of selection of participants | *Methods, Study Population; Results, Participant Enrolment* | Study Population section defines eligibility criteria, exclusions, and consecutive enrolment. |
| Variables | 7 | Clearly define all outcomes, exposures, predictors, potential confounders, and effect modifiers. Give diagnostic criteria, if applicable | *Methods, Brucellosis case classification; Statistical Analysis* | Case classification and Statistical Analysis sections define outcome, predictors, covariates, and diagnostic criteria. |
| Data sources/measurement | 8 | For each variable of interest, give sources of data and details of methods of assessment (measurement) | *Methods, Data Collection; Laboratory Procedures* | Data Collection and Laboratory Procedures sections describe questionnaire data, REDCap capture, specimen handling, and laboratory assays. |
| Bias | 9 | Describe any efforts to address potential sources of bias | *Methods; Discussion, Limitations* | Methods and Discussion address consecutive enrolment, restricted-definition sensitivity analysis, non-enrolment, facility-based sampling, and exposure misclassification. |
| Study size | 10 | Explain how the study size was arrived at | *Results, Participant Enrolment and Study Population* | Results report screened, eligible, enrolled, excluded, and final analytic sample numbers. |
| Quantitative variables | 11 | Explain how quantitative variables were handled in the analyses. If applicable, describe which groupings were chosen and why | *Methods, Statistical Analysis* | Statistical Analysis describes age categories, composite exposure variables, and summary measures. |
| Statistical methods | 12 | (a) Describe all statistical methods, including those used to control for confounding (b) Describe any methods used to examine subgroups and interactions (c) Explain how missing data were addressed (d) If applicable, describe analytical methods taking account of sampling strategy (e) Describe any sensitivity analyses | *Methods, Statistical Analysis* | Statistical Analysis describes prevalence estimation, bivariate testing, modified Poisson regression, covariate selection, diagnostics, missing data handling, and sensitivity analysis. |
| **Results** |  |  |  |  |
| Participants | 13 | (a) Report numbers of individuals at each stage of study (b) Give reasons for non-participation at each stage (c) Consider use of a flow diagram | *Results, Participant Enrolment and Study Population; Fig 2* | Results and Fig 2 report participant flow and reasons for non-enrolment/exclusion. |
| Descriptive data | 14 | (a) Give characteristics of study participants and information on exposures and potential confounders (b) Indicate number of participants with missing data for each variable of interest | *Results, Sociodemographic, Clinical, and Exposure Characteristics; Table 1* | Table 1 presents demographic, clinical, and exposure characteristics by case status. |
| Outcome data | 15 | Report numbers of outcome events or summary measures | *Results, Brucellosis Prevalence* | Results report overall, site-specific, and age-specific brucellosis prevalence. |
| Main results | 16 | (a) Give unadjusted estimates and, if applicable, confounder-adjusted estimates and their precision. Make clear which confounders were adjusted for and why they were included (b) Report category boundaries when continuous variables were categorized (c) If relevant, consider translating estimates into meaningful clinical measures | *Results, Table 1; Multivariable Analysis; Fig 4* | Table 1 and Fig 4 present unadjusted and adjusted prevalence ratios with 95% confidence intervals. |
| Other analyses | 17 | Report other analyses done—e.g., analyses of subgroups and interactions, and sensitivity analyses | *Results, Sensitivity Analysis; Tables S2 and S3* | Sensitivity Analysis and Tables S2–S3 report findings under the restricted case definition. |
| **Discussion** |  |  |  |  |
| Key results | 18 | Summarise key results with reference to study objectives | *Discussion, opening paragraphs* | Discussion summarizes prevalence, site heterogeneity, age-specific findings, and clinical associations. |
| Limitations | 19 | Discuss limitations of the study, taking into account sources of potential bias or imprecision | *Discussion, limitations paragraph* | Discussion addresses cross-sectional design, facility-based sampling, site clustering, non-enrolment, exposure misclassification, case definition, and generalizability. |
| Interpretation | 20 | Give a cautious overall interpretation of results considering objectives, limitations, multiplicity of analyses, results from similar studies, and other relevant evidence | *Discussion* | Discussion interprets age, site, clinical, diagnostic, and exposure findings cautiously in relation to study limitations. |
| Generalisability | 21 | Discuss the generalisability (external validity) of the study results | *Discussion, limitations and conclusion* | Discussion states that findings may not generalize beyond the two ecologically distinct pastoral sites. |
| **Other information** |  |  |  |  |
| Funding | 22 | Give the source of funding and the role of the funders for the present study and, if applicable, for the original study on which the present article is based | *Funding section* | DTRA (HDTRA1-21-1-0041) and NIH (U01AI151799). 'The content is solely the responsibility of the authors and does not necessarily represent the official views of the funders.' |

*Reference: von Elm E, Altman DG, Egger M, Pocock SJ, Gøtzsche PC, Vandenbroucke JP; STROBE Initiative. The Strengthening the Reporting of Observational Studies in Epidemiology (STROBE) statement: guidelines for reporting observational studies. Lancet. 2007;370(9596):1453-7.*
